# Application of an Improved Model for Accident Analysis: A Case Study

**DOI:** 10.3390/ijerph16152756

**Published:** 2019-08-02

**Authors:** Jianhao Wang, Mingwei Yan

**Affiliations:** School of Transportation & Logistics Engineering, Shandong Jiaotong University, Jinan 250357, China

**Keywords:** accident causation model, accident investigation, causes analysis, individual, organization

## Abstract

An improved accident causation model which demonstrates the relationships among different causal factors was proposed in this study. It provides a pathway for accident analysis from the individual level to the organizational level. Unsafe acts and conditions determined by individuals’ poor safety knowledge, low safety awareness, bad safety habits, etc. are the immediate causes of an accident. Deficiencies in safety management systems and safety culture remain the root causes, which can cause consequences at the individual level. Moreover, the weaknesses of an organization’s safety culture can have a great impact on the formation of a good safety climate and can further lead to poor decision-making and implementation of procedures in the safety management system. In order to contribute to a better perception and understanding of the accident causation model, one typical case in the process industry, the oil leak and explosion of the Sinopec Donghuang pipelines, was selected for this study. The causality from immediate causes to root causes is demonstrated in sequence and can be shown in this model explicitly and logically. Several important lessons are summarized from the results and targeted measures can be taken to avoid similar mistakes in the future. This model provides a clear and resourceful method for the safety and risk practitioner’s toolkit in accident investigation and analysis, and the organization can use it as a tool to conduct staff trainings and thus to keep accidents under control.

## 1. Introduction

Accident analysis/investigation is widely recognized as a crucial part of comprehensive and efficient safety management [1]. The investigation report provides details about the occurrence and process of the accident and provides first-hand information for accident analysis and prevention. The best approach to learning about safety is to draw lessons from accidents: it is a great challenge to remember key safety cases so as to avoid unsafe acts in practice since there are endless accident cases, and accompanying causes, throughout the world [2]. Finding out various causes embodied in past cases is of vital importance, and on this basis, feasible mitigation strategies can be made to avoid similar mistakes further. The accident causation model plays an important role in this work by demonstrating the logical relationships among different causal factors, which can help people better understand and remember key lessons easily.

In the past 100 years, numerous accident causation models (theories) were proposed in the domain of safety research, and currently, several typical ones dominate the literature: Greenwood and Woods’s accident-proneness model [3]; Heinrich’s domino-accident causation model [4]; Bird’s loss causation model [5,6]; Rasmussen’s risk management framework (i.e., the AcciMap) [7]; Reason’s omnipresent Swiss Cheese model (SCM) [8]; Wiegmann and Shappell’s human factors analysis and classification system (HFACS) [9,10]; Leveson’s systems theoretic accident modeling and processes model (STAMP) [11,12]. Each accident causation model (theory) engenders its own distinct approach when used for analyzing accidents. Through accurate comparison, their common disadvantage lies in that they fail to define the accident cause and each level so that people may not prevent accidents by directly, accurately, or conveniently applying the analytical processes and interpreting their results [2,13]. 

For example, Greenwood and Woods thought accidents frequently occurred to more accident-prone individuals, but they did not figure out who was accident-prone, so it is difficult to make targeted measures to control people’s behaviors [3]. Heinrich proposed unsafe acts were directly caused by people’s shortcomings which arise from the genetic and socio-environmental factors. However, this model did not give a well-defined connotation of unsafe acts, human shortcomings, genetic factors and their social context, so it is hard to use the hypothesis in practice [4]. Bird believed human shortcomings are formed due to deficiencies in organizational control, but he did not give a definition thereof when an updated domino model was first proposed [5,6]. The SCM observed that unsafe acts are eventually derived from organizational influence factors, but it did not define the organizational factors and the taxonomy requires further work, which makes the hypothesis difficult to apply in practice [8]. Leveson proposed systematic accident analysis methods based on the research of Rasmussen’s high-level functional mechanism and advocated the systematic analysis of the causes of each accident from the top–down (i.e., from a national legislation level to the worker) [11,12]. However, the analytical results and process used are not simple enough to investigate the direct causes of an accident.

Our research team has always been committed to determining how accidents unfold and based on the existing accident causation models (theories) mentioned above, extensive research has been conducted in recent years. We summarized the advantages and disadvantages of those models (theories) and determined the taxonomies and specific contents of various accident causes to further optimize their logical relationships and theoretical framework. As a result, an improved accident causation model and a detailed accident analysis method was proposed [14]. This approach has already been applied in different domains, such as coal mine accidents [15,16,17], hazardous chemical accidents [18,19], aviation accidents [20,21], ferry accidents [22], and construction accidents [23]. The research shows that it can also improve safety in the process industry. The aim of this article is to verify the availability of this model in process safety in an attempt to promote this useful method to analyze and investigate accidents for researchers, practitioners, and investigators. In doing so, an analysis of a recent high-profile incident in the petrochemical domain, the oil leak and explosion of the Sinopec Donghuang pipelines, is presented as a case study.

## 2. Approach to Accident Analysis

**Figure 1:**
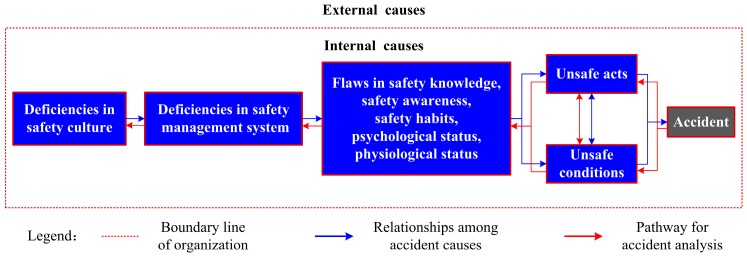
The improved accident causation model and the pathway for accident analysis [2,14,16,18,20].

The improved accident causation model clearly demonstrates the relationship between cause and effect, as shown in Figure 1. It indicates that all accidents belong to the organization and are mainly attributed to internal organizational causes (at both individual and organizational levels) [2]. It is now generally accepted that an accident results from interactions among causal factors residing at all levels of the sociotechnical system, from the government to individuals in the involved organization [7,11]. For the sake of simplicity, causal factors can be classified as “internal causes” and “external causes” based on the manageable boundary of the organization [7,14]. The internal causes are much more changeable and controllable for the managers of the organization to improve safety performance; therefore, they usually serve as the key points for accident analysis. The external causes mainly involve factors from natural events, defective design, poor government supervision, etc., which generally contribute to accidents by influencing the internal causes.

According to Heinrich’s domino theory, unsafe acts and unsafe conditions are the immediate causes of an accident [4]; moreover, mutual impacts between the two also exist. The immediate causes are determined by various factors, mainly including individuals’ safety knowledge (e.g., the theoretical knowledge, operating skill, field experience, etc., stipulated by the organization), safety awareness, and safety habits [24], as well as their psychological and physiological status [25]. It is recognized that errors from the individual level are caused by root causes (i.e., weaknesses in organizational safety management and safety culture) [8,26]. Safety management in an organization is carried out via a safety management system; therefore, deficiencies in the safety management system can be used as indicators to demonstrate the flaws in safety management. The elements of the management system have been introduced in previous literature [20,25,27], and mainly include safety objectives, organizational structure and safety accountabilities, management commitment and responsibilities, hazard identification, risk assessment and mitigation, training and education, resource management, safety communication, continuous monitoring of safety performance, emergency response planning, and continuous improvement. Safety culture, which reflects the beliefs, values, and attitudes shared by the staff related to safety, guides the creation and implementation of the safety management system [25]; thus, poor safety culture or climate in an organization will definitely lead to a deficient safety management system. Safety culture consists of many key elements affecting safety performance [28,29]. This study adopted 32 elements summarized by Fu [20,25,30] and extended from Stewart [31,32], some of which include the importance of safety, importance of a safety management system, economic benefits of safety, role of safety awareness, safety investment, demand for safety training, primary responsibility for safety, safety responsibility of managers, role of safety regulations, and emergency capability.

In Figure 1, the red dotted line is the manageable boundary of an organization related to the accident, which divides all the causes in sociotechnical systems into “internal ones” and “external ones”. The “internal causes” shown in the blue boxes are classified into five categories from individual flaws to organizational deficiencies. The blue arrows indicate the sequence of internal causes leading to an accident, including weaknesses in an organization’s safety culture, deficiencies in the safety management system, flaws in an individual’s safety knowledge, safety awareness, safety habits, etc.; then unsafe acts and unsafe conditions (there is a correlation between the two) eventually lead to an accident. The red arrows indicate the steps for accident analysis, which begins from bad outcomes to immediate causes, to flaws in an employee’s safety knowledge, safety awareness, safety habits, etc., and deficiencies in the organization’s safety management system, and finally to the weaknesses in safety culture. For the sake of application, the consensus process for the analysis of an accident is summarized as follows:

- Performing the events based on the process or timeline and identifying the critical events of the accident further.
- Identifying all organizations (e.g., the design institute, the maintenance unit, the regulators, etc.) related to the accident and figuring out in which one the accident occurred.
- Identifying unsafe acts and unsafe conditions leading to critical events, and then deducing the flaws in individuals’ safety knowledge, safety awareness, safety habits, etc.
- Summarizing the deficiencies in organizational safety management system elements and safety culture elements based on the analysis results at the individual level.
- Performing an analysis of external factors influencing the internal factors or resulting in the accident directly.

## 3. Case Study

To contribute to the understanding of the accident causation model, a typical accident, Qingdao “11·22” oil leak and explosion of the Sinopec Donghuang pipelines, was selected as our case study. Additionally, the availability of the accident analysis method will be demonstrated in this section. Through analysis and discussion, readers will not only understand how and why this disaster occurred, but also remember key points in order to avoid similar mistakes within various industries.

### 3.1. Accident Background

**Figure 2:**
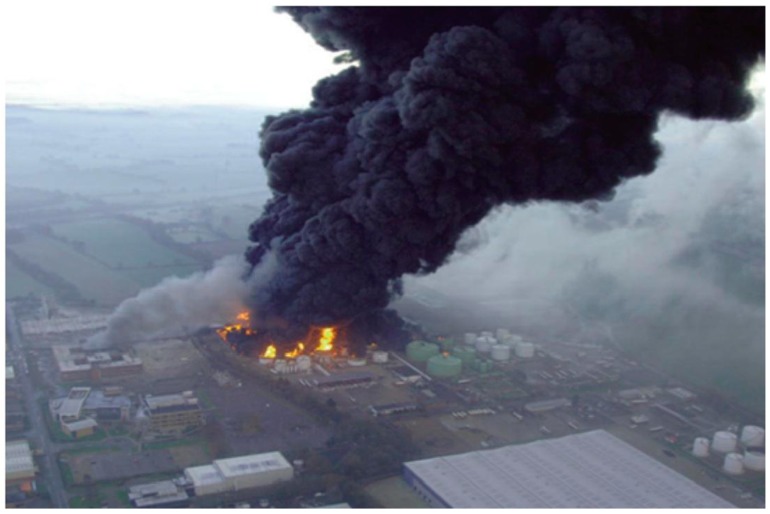
The fire and explosion of the Donghuang oil pipeline.

On 22 November 2013, a devastating oil leak and explosion occurred in Sinopec Donghuang (from Dongying to Huangdao District, Qingdao) pipelines located in Qingdao section, Shandong, China. The catastrophe resulted in many casualties (62 people killed and 136 injured), property loss, and marine pollution, as well as huge negative social impacts and widespread concerns in the process safety field. The appalling fire and explosion scene can be seen in Figure 2. About 40 days after this incident, the former State Administration of Work Safety (SAWS) completed an investigation on it. The report details the occurrence and process of the oil leak and explosion, and the various causes from individuals and organizations, which in combination led to the failure of the system [33].

### 3.2. Process Analysis

**Figure 3:**
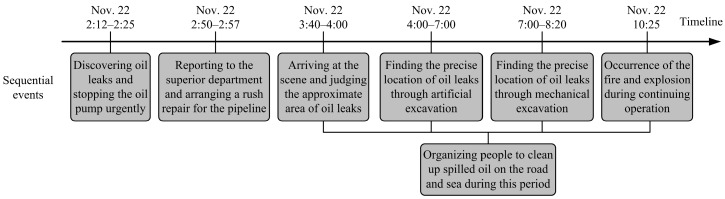
The occurrence and process of the oil leaks and explosion.

An accident is usually caused by sequential occurrences of multiple adverse events. This disaster began with an oil leak that further evolved into fires and explosions. Several sequential events were identified from the investigation report and are shown in Figure 3 based on the timeline. The Donghuang oil pipeline is in total about 248.5 km in length, and the accident section was administrated by the Weifang oil transportation agency affiliated with Sinopec. On 22 November 2013, around 2:12 a.m. (local time), an operator on duty in the Weifang oil transportation agency discovered the pipeline leak through the reduction of the oil pressure. In this urgent situation, the manager stopped the oil pump (at 2:25 a.m.) immediately, and meanwhile, reported and arranged a rush repair. Technicians got to the scene at 3:40 a.m. and began to organize people to excavate after judging the approximate area of the underground broken pipeline. In order to improve the efficiency, excavators and hydraulic hammers were further used for digging and drilling. The precise location of the oil leak was eventually determined at about 8:20 a.m. Due to the serious corrosion of pipelines and large amount of oil leaks, the repair work continued for another two h and the explosion occurred during the process of mechanical excavation (at 10:25 a.m.).

**Figure 4:**
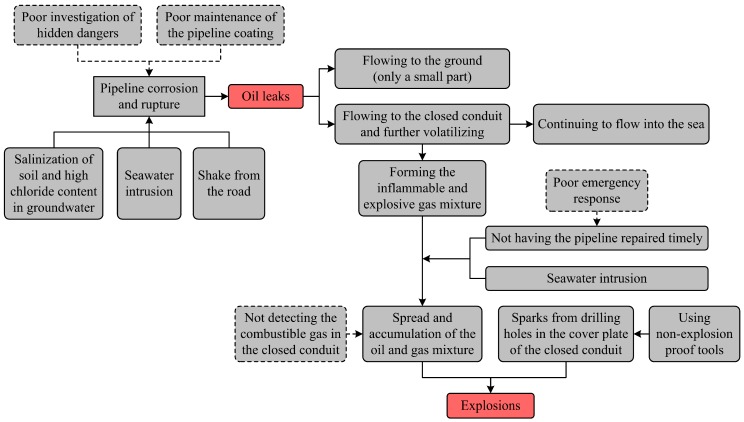
Event sequence diagram (ESD) of the oil leaks and explosion.

According to the timeline, the entire process of this accident can be divided into two critical events: (1) the oil leaks; (2) the explosion. For the sake of better illustration, an event sequence diagram (ESD) was developed (Figure 4). The oil leaked out where the underground pipeline and closed conduit crossed. Seawater intrusion often occurs in the closed conduit due to the tide; this formed an alternate dry–wet and salt-spray environment and caused the salinization of soil and high chloride content in the groundwater. It is no wonder that the pipelines’ corrosion and rupture occurred in these conditions. A large amount of spilled oil flowed to the closed conduit and volatilized gradually, thus forming the inflammable and explosive gas mixture. When the rush repair was underway, the oil and gas mixture, affected by the seawater intrusion, spread and accumulated quickly in this space.

It should be noted more than eight h passed after the leaks were discovered, but repair of the broken pipelines was not completed because of poor emergency disposal. According to estimation, the total amount of oil leakage from the ground, closed conduit, and sea was up to 2000 tons during this period. However, people on site were not aware of the risk and still used non-explosion-proof tools to open the cover plate of the closed conduit without taking protective measures. The oil and gas mixture ignited and detonated from the exposed sparks of the mechanical drilling. 

**Figure 5:**
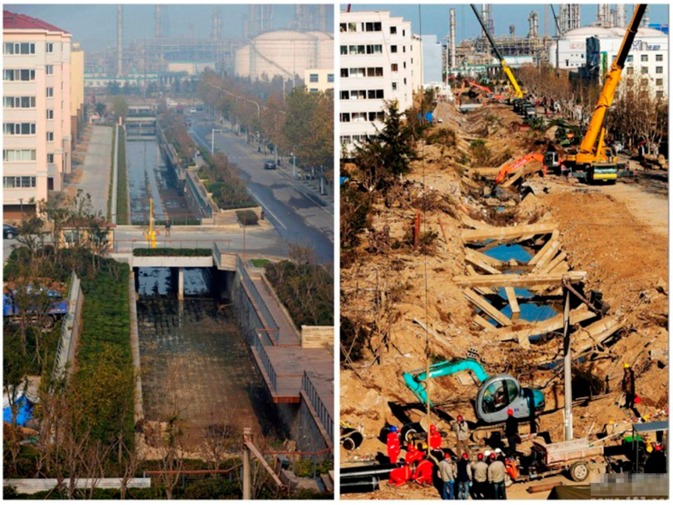
Compared accident scenes of the present (**left**) and past (**right**).

The explosion seriously destroyed the block and caused extensive marine pollution. The damaged pipelines located in the dangerous section were discontinued and the closed conduit was transformed into an open one for the sightseeing and warning. The compared accident scenes of the present and past can be seen in Figure 5.

### 3.3. Causes Analysis

Through the process analysis above, two critical events (i.e., the oil leaks and the explosion) have been identified from this disaster and the cause analysis will be centered on them. There are large differences between the causes of the two events, especially in respect to individual causes; therefore, the analysis results are shown in separate models for the sake of clear and logical illustration. 

**Figure 6:**
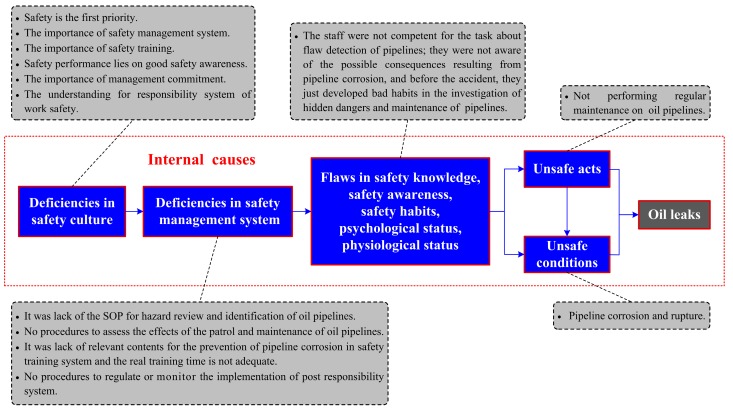
Causes analysis of oil leaks based on the accident causation model. SOP: standard operating procedure.

**Figure 7:**
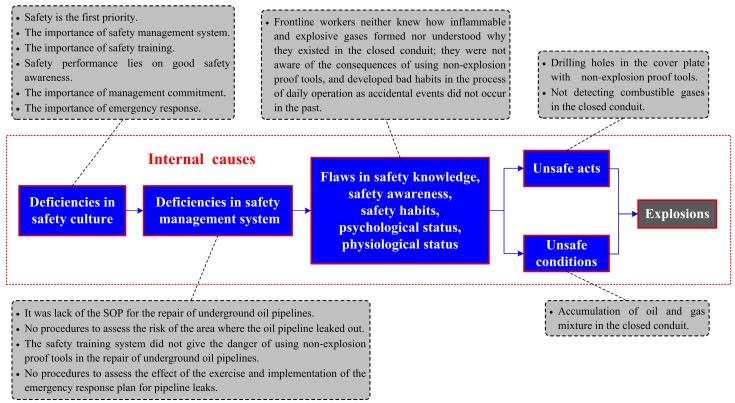
Causes analysis of explosions based on the accident causation model.

The accident section of the Donghuang oil pipeline is administrated by Weifang oil transportation agency, and the on-site personnel such as the management, repairmen, excavator operators, etc. are all employed by this unit. Therefore, Weifang oil transportation agency should take full responsibility for the accident. Causes in relation to this organization, usually considered as the internal ones, are of crucial importance. Additionally, this accident involved several local government agencies (e.g., the supervision department of work safety, the planning and design department, the office of emergency management, etc.) whose faults could also have contributed to the oil leaks and explosion. The internal causes illustrated in the improved accident causation model are shown in Figure 6 and Figure 7, respectively.

#### 3.3.1. The Oil Leaks

The corrosion and rupture of the pipeline, which was considered to be the unsafe condition cause, directly led to the oil leaks. The pipelines corroded easily due to the effect of bad local hydrogeological conditions such as the salt-spray environment, salinization of soil, and high content of chloride in the groundwater. The *Oil and Gas Pipelines Protection Law of the People’s Republic of China* (PRC) (article 23) stipulates that pipeline enterprises shall conduct regular detection and maintenance of pipelines to ensure that they are in good condition; the sections and sites with high risks shall be monitored in a critical manner, and effective measures shall be taken to prevent pipeline accidents. However, employees in the Weifang oil transportation agency did not do this work well according to the rules, and the coating overhaul for the Donghuang oil pipelines that began in 2011 had not yet been finished before the incident. The organization’s nonfeasance caused the corrosion and rupture to exist for a long time, and we believe it caused the unsafe condition indirectly. Thus, this also provides an effective way to prevent some unsafe conditions, namely by turning to the elimination of corresponding unsafe acts. 

The flaws in individuals’ safety knowledge, safety awareness, and safety habits resulted in the unsafe acts directly. As the investigation noted, Weifang oil transportation carried out a total of three flaw detections on the Donghuang oil pipelines during 2009–2013, but the staff in charge of the work failed to find the pipeline’s corrosion and rupture during their inspections; therefore they might not be qualified for the technical task because of their lack of knowledge and experience. All members in the organization should attach importance to their job responsibilities, especially managers, whose poor inspection and supervision remain very important factors for the occurrence of unsafe acts. However, people who were in charge of the regular maintenance of equipment and facilities did not abide by the rules and neglected the overhaul of the pipelines in production. Based on this, we can deduce that the staff were neither aware of the adverse consequences resulting from pipeline corrosion nor had good habits to carry out the regular maintenance of the pipeline. 

The roots of an accident lie in the errors of an organization; moreover, individual behaviors are largely affected by organizational factors [8]. Therefore, the deficiencies in an organizational safety management system and safety culture could be inferred according to the above analysis on individual flaws. 

According to the *Safety Specification for Crude Oil and Natural Gas Pipelines* (article 8), several deficient elements, such as hazard identification, safety training and education, safety accountability, and equipment management (i.e., the maintenance and detection for oil pipelines), were identified from the safety management system of the Weifang oil transportation agency. The lack of a standard operating procedure (SOP) for hazard identification led to the staff’s inadequate training and insufficient knowledge. This made them fail to identify the pipeline’s hidden dangers in the process of past flaw detections (a total of three times in 2009, 2011, and 2013). Before this accident, the on-site staff had already performed regular patrol and maintenance for the oil pipelines, but the corrosion and rupture were not prevented effectively because there was no procedure or process in the safety management system to assess the effect of the implementation of engineering standards. Safety training, as one of the most important means for accident prevention, was not well conducted in the Weifang oil transportation agency. There were no adequate theoretical contents about the prevention of pipeline corrosion in the training document. Some operating skills about the pipelines’ detection were not trained well in accordance with engineering standards; moreover, the specified training time for employed front-line workers was not sufficient. The flawed organizational roles caused the ambiguous assignment of responsibilities for the protection of operational safety within the Weifang oil transportation agency. The violation of regulations reflected that the post responsibility system was not valued by the operators and managers, and the organization did not establish processes to monitor its daily implementation either. 

The deficient safety management system indicated that members in the organization did not reach an agreement on safety beliefs such as “safety is the first priority”, “the importance of safety management system”, “the importance of safety training”, “the importance of laws and engineering standards” or “safety performance depends on good safety awareness”. Good leadership can contribute to a good safety climate. The key role for leaders and managers is to develop and drive a culture for safety management within the organization. The staff in the Weifang oil transportation agency ignored that safety should be put first in daily work and they did not pay much attention to the roles of safety awareness and the responsibility system for accident prevention. Moreover, the top management failed to demonstrate their commitment to support the implementation and audit of the safety management system. Collectively, managers and leaders should provide the organizational systems and drive the organizational culture that determines not only what people in the organization do, but more importantly, how they do it.

In addition, there were several external factors related to the occurrence of the oil leaks. The local supervision department of work safety who is the lead unit for the protection of underground pipelines failed to perform its supervision duties well because the coating overhaul for the Donghuang oil pipelines was not finished within two years. Moreover, it failed to urge the pipeline enterprise to carry out the hazard identification and regular maintenance for oil pipelines and did not realize the closed-loop management of the safety inspection through the form of “reviewing”. There was also some irrationality in the layout of the oil pipelines approved by the local planning and design department, which had a great impact on the occurrence of the oil leaks. As mentioned above, the oil pipeline around the accident site was installed in a closed conduit, which posed a higher risk when the leaked oil easily flowed into the conduit and caused inconvenience for the maintenance and rush repair of the installation. In view of this, the damaged pipelines located in the dangerous section are now out of use.

#### 3.3.2. The Explosions

Another two unsafe acts made the oil leaks further evolve into multiple explosions and large-scale fires. During the rush repair, the closed conduit was full of inflammable and explosive mixtures of oil and gas due to the seawater intrusion, thus causing another dangerous condition. According to the *Oil and Gas Pipelines Protection Law of the PRC* (article 30), it is forbidden to use mechanical tools for excavation and construction within five meters on both sides of the center line of the pipeline. The spark, a requirement for this accidental event, was just generated from drilling holes in the cover plate with a non-explosion-proof hydraulic hammer. If on-site staff performed effective gas detection for the closed conduit in accordance with the operating procedures stipulated in *Safety Specification for Crude Oil and Natural Gas Pipelines* (article 8.4) before the excavation, this explosion could have been avoided. Indeed, this disaster was classified as an “accountability accident” by the SAWS and almost all unsafe acts violated regulations or engineering standards; therefore, identifying and eliminating those specific unsafe acts is of crucial importance for the prevention of such similar mistakes.

Safe acts or unsafe acts, by the accident causation model, are directly determined by individuals’ habitual behaviors such as competence, awareness, thought, habit, etc. The two unsafe acts above were produced by the staff involved in the oil pipeline’s rush repair. As the investigation report noted, the management and employed front-line workers at the scene were not trained adequately and lacked the experience to rush repair the underground pipelines. They did not know how inflammable and explosive gases formed, nor did they understand why the mixture spread and accumulated in the closed conduit. According to the survey, most people did not even have the theoretical knowledge about the chemical property of crude oil and did not think that it could be easily detonated like refined oil (e.g., gasoline, diesel oil, etc.). Also, the staff was not aware of the consequences of using non-explosion-proof tools and developed bad habits in the process of daily operations since accidental events had not occurred in the past. It is therefore no wonder that unsafe acts appeared in this event.

The poor disposal of the oil leak indicates that there are also lots of flaws in some elements of the Weifang oil transportation agency’s safety management system, such as equipment management (i.e., the regulation for repair), risk assessment and mitigation, safety training and education, emergency response planning, etc. The repair of underground oil pipelines is a high-risk task and the organization must establish a comprehensive SOP in the management system according to related engineering standards for managers and operators. The personnel allocation (operators and supervisors), tools selection, job steps, and protective measures should be detailed in this procedure. However, the Weifang oil transportation agency was not aware of the risks in the repair of leaked oil pipelines and neglected the importance of safety procedures. This also caused deficiencies in other systems or plans. Some contents about the SOP, especially the danger of using non-explosion-proof tools in the repair of oil pipelines, were not given in the safety training system, and the occurrence of the explosions also had a lot to do with peoples’ lack of knowledge and bad treatment in the rush repair. As we know, the leaks of oil pipelines can easily trigger domino events once disposed improperly. According to the survey, there was no procedure or process in the safety management system to assess the risk of the area where the pipeline leaked, and protective measures were not taken by the organization, either. Additionally, emergency plans about the pipeline leaks need to be mastered and exercised by all concerned but the management failed to ensure that emergency training was provided as intended. According to the investigation, the regular exercise for pipeline leaks was actually carried out by the Weifang oil transportation agency, but there was no procedure or process in the safety management system to assess the effect of its implementation and the emergency response plan was not performed and audited all the time.

There are no significant differences in the deficiencies of organizational safety culture elements between the analyses of the two critical events; thus, analyses in this section will be simplified. The occurrence of explosions indicated that the members’ inadequate consensus on safety beliefs such as “safety is the first priority”, “the importance of safety management system”, “the importance of safety training”, “safety performance lies on good safety awareness”, “the importance of laws and engineering standards”, etc. could be inferred as poor safety culture. The quality of leadership and commitment to safety can drive or limit the safety culture of an organization. However, the leaders and managers in the Weifang oil transportation agency failed to deeply understand the process safety management (PSM) program—its importance for maintaining both safe operations and compliance, its key roles and responsibilities, as well as current issues and challenges the organization faced in its implementation. As mentioned above, improper disposal of the oil leaks triggered the multiple explosions; clearly, Weifang oil transportation agency neglected the importance of emergency management for the PSM. The management should promote all staff to master the emergency plan and supervise its implementation and regular audit.

In addition, there were several external factors contributing to the explosion and serious casualties. The local administration committee of the development zone failed to fully understand the severity of the crude oil leakage and initially classified it as a general emergency (total four levels: particularly serious, serious, major, and general) based only on the report of the pipeline enterprise. This led to a poor command and coordination for the emergency: warning and road closure measures were not taken on the site; the nearby masses were not notified and evacuated in a timely manner; and problems such as violations of regulations from on-site emergency personnel were not found and stopped. Moreover, the local office of emergency management did not organize experts to carry out the research and assessment on the development trend of the oil leaks and thus failed to raise the level of the emergency response timely. Therefore, it was not surprising that the multiple explosions occurred, and the casualties and property losses were so severe.

## 4. Conclusions

This article presents an improved accident causation model and its application in accident analysis through a specific case, the oil leaks and explosion of the Sinopec Donghuang pipelines. The main purpose of this study was to generalize a universal method for accident analysis, and meanwhile, it is significant to help people learn lessons from this catastrophic event through this model.

The accident causation model provides a pathway for accident analysis from the individual level to the organizational level. A timeline of events is a primary step for the task and further determining the critical ones is of vital importance. For every critical event, unsafe acts and unsafe conditions, which are considered as the immediate causes, should be identified first. On this basis, unsafe behaviors (i.e., individuals’ safety knowledge, safety awareness, safety habit, psychological status, and physiological status), deficiencies in organizational safety management system, and safety culture elements can be further deduced one by one. Finally, external factors influencing the occurrence of the events could be determined in detail.

In this study, the accident was divided into two sequential events (i.e., the oil leaks and explosion), and causal factors involved in each one were analyzed by this model. Thus, several important lessons for the PSM can be summarized as follows.

- Front-line workers’ unsafe acts or nonfeasance often results in the facility’s unsafe conditions, which may spawn severe consequences directly. Thus, it is necessary for the management to strengthen the field supervision and inspection.
- Adequate safety training and education for individuals is conductive to improving their safety knowledge, safety awareness, safety habit, or even psychological status, and most unsafe acts may be avoided with it.
- The SOPs for some dangerous works, such as hazard identification, flaws detection, emergency disposal, etc. are of the essence, and procedures for the monitoring of their implementation effects should be given at the same time.
- Periodic safety audits must be performed with rigor to identify the weaknesses of the safety management system before an incident occurs. If any issues were found, all should be reported and corrected.
- The quality of leadership and commitment to safety can drive or limit the safety climate of an organization. Managers must develop and sustain a sound culture that embraces both process safety and occupational safety.
- External causes from other organizations, such as unreasonable design and planning, inadequate supervision and inspection, poor command and coordination, etc., also had significant impacts on the occurrence and development of the accident. The government agencies shall perform their duties well.

Admittedly, the accident causation model is not perfect and still needs to be improved constantly. The present study intends to promote a universal method for accident analysis, especially in the process safety domain, but it is difficult to offer a convincing fact with only one case study. Thus, continued research needs to be carried out in the future.

## References

[1] Abdolhamidzadeh B., Hassan C.R.C., Hamid M.D., FarrokhMehr S., Badri N., Rashtchian D. (2012). Anatomy of a domino accident: Roots, triggers and lessons learnt. Process. Saf. Environ. Prot..

[2] Fu G., Chen P., Zhao Z., Li R. (2019). Safety Is About Doing the Right Thing. Process Saf. Prog..

[3] Greenwood M., Woods H.M. (1919). The Incidence of Industrial Accidents upon Individuals with Specific Reference to Multiple Accidents.

[4] Heinrich H.W., Petersen D., Roos N.R. (1980). Industrial Accident Prevention: A Safety Management Approach.

[5] Bird F.E.J., Germain G.L. (1985). Practical Loss Control Leadership.

[6] Bird F.E.J., Germain G.L., Clark D.M. (2003). Practical Loss Control Leadership.

[7] Rasmussen J. (1997). Risk management in a dynamic society: A modelling problem. Saf. Sci..

[8] Reason J. (1990). Human Error.

[9] Wiegmann D.A., Shappell S.A. (2003). A Human Error Approach to Aviation Accident Analysis–The Human Factors Analysis and Classification System.

[10] Patterson J.M., Shappell S.A. (2010). Operator error and system deficiencies: Analysis of 508 mining incidents and accidents from Queensland, Australia using HFACS. Accid. Anal. Prev..

[11] Leveson N.G. (2004). A new accident model for engineering safer systems. Saf. Sci..

[12] Leveson N.G. (2011). Applying systems thinking to analyze and learn from events. Saf. Sci..

[13] Salmon P.M., Cornelissen M., Trotter M.J. (2012). Systems-based accident analysis methods: A comparison of Accimap, HFACS, and STAMP. Saf. Sci..

[14] Fu G., Fan Y., Tong R., Gong Y. (2016). A Universal Methodology for the Causation Analysis of Accidents (4th Edition). J. Accid. Prev..

[15] Fu G., Yin W., Dong J., Di F. (2013). Behavior-based accident causation: The 24Model and its safety implication in coal mines. J. China Coal Soc..

[16] Fu G., Zhao Z., Hao C., Wu Q. (2019). The Accident Path of Coal Mine Gas Explosion Based on 24 Model: A Case Study of the Ruizhiyuan Gas Explosion Accident. Processes.

[17] Wang J., Zhang J., Zhu K., Zhou L. (2016). Anatomy of explosives spontaneous combustion accidents in the Chinese underground coal mine: Causes and prevention. Process. Saf. Prog..

[18] Fu G., Zhou L., Wang J., Shi M. (2018). Analysis of an explosion accident at Dangyang Power Plant in Hubei, China: Causes and lessons learned. Saf. Sci..

[19] Fu G., Wang J., Yan M. (2016). Anatomy of Tianjin Port fire and explosion: Process and causes. Process Saf. Prog..

[20] Xue Y., Fu G. (2018). A modified accident analysis and investigation model for the general aviation industry: Emphasizing on human and organizational factors. J. Saf. Res..

[21] Zhang Z., Li H., Gao K., Ding W., Duo W. (2016). Research on the behavior causes of Yichun “8-24” catastrophic accident. Saf. Sci. Study.

[22] Suo X., Fu G., Wang C., Jia Q. (2017). An Application of 24 Model to Analysis Capsizing of the Eastern Star Ferry. Pol. Marit. Res..

[23] Zhang H., Gong Y., Fu G. (2017). Causes classification and statistical analysis on falling accidents on construction sites based on “2-4” model. J. Saf. Sci. Technol..

[24] Fu G., Lu B., Chen X. (2005). Behavior Based Model for Organizational Safety Management. China Saf. Sci. J..

[25] Fu G. (2013). Safety Management–A Behavior-based Approach to Accident Prevention.

[26] Broadribb M.P. (2015). What have we really learned? Twenty five years after piper alpha. Process Saf. Prog..

[27] Zhou L. (2018). Safety Management System Failures of Chinese Hazardous Chemical Accidents.

[28] Hale A.R. (2000). Culture’s confusions. Saf. Sci..

[29] Mearns K., Kirwan B., Reader T.W., Jackson J., Kennedy R., Gordon R. (2013). Development of a methodology for understanding and enhancing safety culture in Air Traffic Management. Saf. Sci..

[30] Ma Y. (2017). Study on the Construction Method of Enterprise Safety Culture.

[31] Stewart J.M. (2002). Managing for World Class Safety.

[32] Stewart J.M. (2011). The turn-around in safety at the Kenora pulp paper mill. Prof. Saf..

[33] State Administration of Work Safety Accident Investigation Report of Qingdao ‘11-22’ Oil Leaks and Explosions of Sinopec Donghuang Pipelines. http://www.chinasafety.gov.cn/gk/sgcc/tbzdsgdcbg/2013/201306/t20130626_245228.shtml.

